# New Insight in Loss of Gut Barrier during Major Non-Abdominal Surgery

**DOI:** 10.1371/journal.pone.0003954

**Published:** 2008-12-17

**Authors:** Joep P. M. Derikx, Dick A. van Waardenburg, Geertje Thuijls, Henriëtte M. Willigers, Marianne Koenraads, Annemarie A. van Bijnen, Erik Heineman, Martijn Poeze, Ton Ambergen, André van Ooij, Lodewijk W. van Rhijn, Wim A. Buurman

**Affiliations:** 1 Department of Surgery, University Hospital Maastricht & Nutrition and Toxicology Research Institute (NUTRIM), Maastricht University, Maastricht, the Netherlands; 2 Department of Paediatrics, University Hospital Maastricht, Maastricht, the Netherlands; 3 Department of Anaesthesiology, University Hospital Maastricht, Maastricht, the Netherlands; 4 Department of Methodology and Statistics, University of Maastricht, Maastricht, the Netherlands; 5 Department of Orthopaedic Surgery, University Hospital Maastricht, Maastricht, the Netherlands; University of Giessen Lung Center, Germany

## Abstract

**Background:**

Gut barrier loss has been implicated as a critical event in the occurrence of postoperative complications. We aimed to study the development of gut barrier loss in patients undergoing major non-abdominal surgery.

**Methodology/Principal Findings:**

Twenty consecutive children undergoing spinal fusion surgery were included. This kind of surgery is characterized by long operation time, significant blood loss, prolonged systemic hypotension, without directly leading to compromise of the intestines by intestinal manipulation or use of extracorporeal circulation. Blood was collected preoperatively, every two hours during surgery and 2, 4, 15 and 24 hours postoperatively. Gut mucosal barrier was assessed by plasma markers for enterocyte damage (I-FABP, I-BABP) and urinary presence of tight junction protein claudin-3. Intestinal mucosal perfusion was measured by gastric tonometry (P_r_CO_2_, P_r-a_CO_2_-gap). Plasma concentration of I-FABP, I-BABP and urinary expression of claudin-3 increased rapidly and significantly after the onset of surgery in most children. Postoperatively, all markers decreased promptly towards baseline values together with normalisation of MAP. Plasma levels of I-FABP, I-BABP were significantly negatively correlated with MAP at ½ hour before blood sampling (−0.726 (p<0.001), −0.483 (P<0.001), respectively). Furthermore, circulating I-FABP correlated with gastric mucosal P_r_CO_2_, P_r-a_CO_2_-gap measured at the same time points (0.553 (p = 0.040), 0.585 (p = 0.028), respectively).

**Conclusions/Significance:**

This study shows the development of gut barrier loss in children undergoing major non-abdominal surgery, which is related to preceding hypotension and mesenterial hypoperfusion. These data shed new light on the potential role of peroperative circulatory perturbation and intestinal barrier loss.

## Introduction

Patients undergoing major surgery or sustaining severe trauma are at risk of developing morbidity and mortality from postoperative or posttraumatic systemic inflammatory response syndrome (SIRS), sepsis and multiple organ failure (MOF). The development of such potentially lethal complications in relatively healthy surgical or trauma patients is poorly understood [1], [2]. Moreover, few human studies investigated the hypothesis, generated from animal studies, that the intestines are central in the origin of postoperative and posttraumatic sequelae [3]–[5]. Major surgery accompanied by systemic hypotension and blood loss is thought to lead to redistribution of blood to preserve the vital organs (brain and heart) at the expense of the splanchnic circulation [3]–[5]. Low mesenteric blood flow subsequently leads to injury of the cells at the most distal point from the mucosal blood supply, being the mature enterocytes [6].

Experimental animal models, resembling the clinical situation of major surgery and trauma, show that haemorrhagic shock leads to disruption of the gut barrier, measured by elevated circulating levels of Fatty Acid Binding Proteins (FABP), originating from damaged intestinal epithelial cells and derangement of tight-junctions [7], [8]. Moreover, translocation of macromolecules, microbial products and microbiota from the intestinal lumen to the circulation and mesenteric lymph nodes, spleen and liver occur [5], [7]. The inflammatory response to translocated microbial products as endotoxin has been reported to be induced via various innate immune mechanisms, ranging from Toll Like Receptors to complement activation [9], [10].

Studies in patients undergoing major gastro-intestinal, cardiac or vascular surgery, investigating the role of the gut in the development of postoperative complications, are largely restricted to data on increased intestinal permeability for sugars, ^51^Cr-EDTA and the circulatory levels of endotoxin [11]–[19]. Several authors report changes in these parameters in patients following major surgery, indicating that the gut barrier is injured [11]–[14]. However, other reports using these tests lack to support these data [15]–[18]. Moreover, the value of measuring gut barrier with the use of sugar absorption probes is argued [19]. In conclusion, the debate regarding the involvement of the gut in patients undergoing major surgery is still open.

Plasma and urinary markers are currently available as useful non-invasive tools to study the condition of enterocytes and tight-junctions (TJ), the two components comprising the gut mucosal barrier. Enterocyte damage was assessed using plasma levels of Intestinal-Fatty Acid Binding Protein (I-FABP), a small cytosolic, water-soluble protein, primarily limited to mature enterocytes of small and large intestine [20]. I-FABP plasma levels rise rapidly after episodes of acute intestinal ischaemia and inflammation [20]–[22]. Next, also circulating levels of Ileal-Bile Acid Binding Protein (I-BABP), which is exclusively present in mature enterocytes of the jejunum and ileum, were assessed [23]. TJ between neighbouring enterocytes are an important constituent of the intestinal epithelial barrier [24]. The transmembrane TJ protein Claudin-3, the essential sealing protein, disappears rapidly from the TJ following haemorrhagic shock and is released into the urine (own unpublished data).

The principal aim of this study was to investigate whether major non-abdominal surgery leads to intestinal barrier loss.

## Methods

### Study design and patients

This is a prospective clinical observational study in children undergoing spinal fusion surgery because of scoliosis in the University Hospital Maastricht between March 2006 and October 2007. Major spinal fusion surgery is characterized by long operation time, significant blood loss, prolonged systemic hypotension and the potential development of postoperative complications [25]. This type of surgery was chosen because it does not directly compromise the intestines by intestinal manipulation or the use of extracorporeal circulation [25].

Informed, written consent was obtained by all patients or both parents/caretakers whose information was used in the study prior to inclusion; the study was conducted with approval from the local medical ethical committee.

### Surgical procedures

#### Preoperative preparation and anaesthesia

Anaesthesia was induced and maintained with either a volatile based technique with sevoflurane or an intravenous technique with propofol, combined with an opioid and a non-depolarising muscle relaxant. Sensory evoked potentials were monitored in patients at risk for spinal cord problems during surgery. According to the hospital protocol an intravenous technique with propofol was used in these patients. All patients were intubated and ventilator settings were adjusted to obtain normocapnia. Each patient had a forced-air warming system and all intravenous fluids were warmed to prevent hypothermia.

In addition to standard monitoring an arterial line was inserted into a radial artery to measure arterial pressure and to sample arterial blood, and a catheter was introduced into the bladder to measure urine output. Blood-soaked gauzes were weighted as they were passed off the surgical field and the blood content of the cell saver was measured to measure blood loss.

Perioperative fluid therapy was adapted to the individual patient with the aim to keep the patient normovolaemic throughout the operation. Isotonic crystalloids were used for maintenance and third space losses. Blood loss was replaced 1∶1 with blood or colloid or 3∶1 with crystalloids. Fluid administration was guided by calculation of maintenance and third space losses, blood loss, the arterial blood pressure, and haemoglobin values. There was no protocol to keep the blood pressure above or below a certain value.

#### Surgery

All the operations were performed by 2 senior spine surgeons (LvR and AvO) using three fusion approaches: posterior spinal fusion (PSF), anterior spinal fusion (ASF), and combined anterior and posterior fusion. The decision regarding the preferred fusion was made based on curve location, aetiology, rigidity, and the child's age, according to the current standards of scoliosis operative repair.

In PSF, the patient was positioned prone on padded chest rolls, rolled blanket bolster, or a Wilson spinal frame to provide adequate cushioning for the chest and abdomen while allowing vacant space preventing abdominal pressure. The skin was incised in a straight line over the vertebrae to be fused. Following osteotomy of all the spinous processes and facets included at the fusion area, the vertebrae were instrumented with combinations of pedicle-screws and hooks (CD Horizon Legacy 5.5 or 4.5 spinal systems, Medtronic, Heerlen, the Netherlands). Curve correction was performed with a combination of derotation and compression–distraction manoeuvres, and, if necessary, also by in situ bending of the rods. In ASF, the patient was placed on the operating table in the lateral position. The approach is from the convex scoliotic side. A thoracoabdominal approach, which included a split of the diaphragm near its insertion, retroperitoneal approach, or lateral intrathoracic approach was performed through the side of the curve convexity, with the patient lying on his side. Following exposure of the vertebrae, the involved discs and ribs were excised and the segmental vessels ligated or preserved. In all cases of combined fusion, single-staged procedures were carried out.

CD Horizon Legacy 5.5 or 4.5 spinal systems were used depending on age and weight of the patient. In case of anterior instrumentation CD Horizon Eclipse spinal system was used (Medtronic). No drains were used.

#### Postoperative care

At the end of the surgery, all the children were transferred to the paediatric Intensive Care Unit (ICU). Extubation was performed after stabilization of vital signs and according to accepted weaning parameters (usually 4–6 hours after surgery). Paediatric ICU management was provided by the attending physicians guided by the same general management strategy and consisted of intravenous fluid administration, correction of hypovolaemia, electrolyte disturbances and/or anaemia and analgesics (acetaminophen and morphine). Antibiotic cover (amoxicillin with clavulanate) was given starting preoperatively until 24 hours after the end of surgery. Oral feeding was introduced the day after surgery.

Follow-up in the ICU included a daily physical examination, vital signs monitoring, routine blood tests, and chest radiographs or other ancillary tests as required. The attending physicians recorded complications and events.

### Blood and urine sampling

Blood samples were collected from the arterial line in pre-chilled EDTA vacuum tubes (BD vacutainer, Becton Dickinson Diagnostics, Aalst, Belgium) and kept on ice. Blood was centrifuged at 4°C, 4000×G for 15 minutes. Plasma was immediately stored in aliquots at −80°C until analysis. Blood was sampled before surgery (after the induction of anaesthesia), at 2 hours intervals during surgery and 2, 4, 15 and 24 hours postoperatively from the arterial line.

Fresh specimens of urine were collected from the urinary bladder catheter, kept on ice and then frozen at −80°C in aliquots within 2 hours of collection. Urine was collected every 20 minutes in the first 2 hours during surgery and thereafter at the same moments as blood was sampled.

### Measurements of FABP and claudin-3

Plasma concentrations of I-FABP were determined using a highly specific commercially available enzyme-linked immunosorbent assay (ELISA) that selectively detects human I-FABP (standard: 20–5,000 pg/ml), kindly provided by Hycult Biotechnology (Uden, the Netherlands) and I-BABP (standard: 0.32–5 ng/ml) as previously described [26].

Claudin-3 urine levels were analyzed by western blotting. Equal amounts of each sample (adjusted to urinary creatinine levels) were separated by SDS-PAGE gel, transferred to PVDF-membrane and probed using primary antibody to claudin-3 (Rabbit anti-claudin-3 (34–1700), Zymed Laboratories, San Francisco, CA). After incubation with goat anti rabbit HRP-conjugated secondary antibody (Jackson, West Grove, PA), signal was detected by supersignal west pico chemiluminescence substrate (Pierce, Etten-Leur, the Netherlands). Band intensity was semi-quantitatively analyzed using Quantity One (Biorad, Hercules, CA).

### Measurements of gastric mucosal tonometry

A gastric tonometry catheter (14F, Datex Ohmeda, Helsinki, Finland) was introduced for measurement of intramucosal carbon dioxide pressure (P_r_CO_2_ in kPa) throughout the surgical procedure in the last seven patients, using the gas-automated capnograph (Tonocap TC-200, Datex-Ohmeda).

Gastric tonometry measurements (P_r_CO_2_, and mucosal-arterial pCO_2_ gap (P_r-a_CO_2_-gap)) were measured at 30-minute intervals from the start until the end of surgery using gas-automated capnography. The pCO_2_ values of the blood gases were corrected for the central blood temperature measurements, using the formulas provided by the manufacturer (ABL 100, Radiometer, Copenhagen, Denmark).

### Statistical analysis

Statistical analysis was performed with Prism 4.0 for Windows (GraphPad Software Inc. San Diego, CA) and SPSS 15.0 (SPSS, Inc., Chicago, IL). Plasma FABP concentrations were presented as mean±standard error (SEM). Normality of all data obtained was verified by Kolmogorov-Smirnov test.

Linear mixed model regression was used to analyze changes over time in plasma FABP levels. Within-person correlations between mean arterial pressure (MAP), P_r_CO_2_/CO_2_-gap and circulating levels of FABP at all studied time points were computed. Mixed model analysis accounts for unbalanced numbers of samples measured, because at some time points not all samples could be obtained because of removal of the arterial line. Differences in plasma FABP levels between individual time periods were compared using t tests of the mixed model procedure. Next, to characterize the total amount of intestinal mucosal cell injury during surgery, area under the curve (AUC) for I-FABP and I-BABP was calculated for each patient. The AUC_I-FABP_ and AUC_I-BABP_ for patients with and without early complications was compared using unpaired t test. Early complications were defined as complications occurring in the intraoperative or initial hospitalization period.

A p-value below 0.05 was considered to be statistically significant.

## Results

### Patients

Twenty patients undergoing spinal fusion surgery were consecutively included in the study, 15 girls and 5 boys. Median age was 12 years (range: 2–16 years). Demographic, surgical and fluid balance data are presented in Table 1. Intraoperative fluid resuscitation was adequate as evidenced by; 1) a mean (SEM) positive fluid balance (total fluid in minus blood loss) of 13 (1) ml/kg/hr; 2) adequate diuresis; 3) low plasma lactate levels and; 4) adequate plasma haemoglobin value (data not shown).

**Table 1:** Demographic, surgical and fluid balance characteristics.

No^0^	Age (y)	Weight (kg)	Surgery^1^	Duration surgery (hr)	History^2^	Early complications^3^	Blood loss (ml/kg)^4^	Fluid in (ml/kg)^4^	Diuresis (ml/kg/hr)^4^	Mean (range) MAP (mmHg)^4^	Mean (range) lactate (mmol/l)^4^
1	8	36	ASF T7-L4	6	spina bifida, hydrocephalus	fever, UTI	14	103	3.5	57 (52–63)	1.0 (0.8–1.2)
2	15	40	ASF+PSF T3-S1	8	cerebral palsy, spastic diplegia, IVH		20	131	1.7	54 (41–66)	1.2 (1.0–1.3)
3	12	51	ASF+PSF T12-L3	8	-	fever, pneumonia	59	163	1.1	56 (50–66)	1.9 (1.6–2.6)
4	12	31	ASF+PSF T2-S1	9	DiGeorge syndrome; vascular spinal cord lesion	melaena	32	190	1.5	59 (45–77)	1.6 (0.8–1.9)
5	13	59	PSF T4-L3	6	cleft lip nose deformity	-	53	156	2.5	65 (56–70)	1.3 (1.2–1.4)
6	11	37	PSF T3-T12	4	spina bifida occulta	-	11	95	1.5	81 (70–95)	1.8 (1.6–2.1)
7	8	45	PSF T2-L5	4	spinal muscular atrophy	-	14	68	1.9	55 (51–60)	0.9 (0.7–1.1)
8	10	48	ASF T6-T12	4	-	-	13	73	4.2	66 (59–75)	1.5 (1.2–1.9)
9	14	33	PSF T3-S1	8	spastic tetraplegia, panencephalitis	-	91	341	5.3	65 (51–80)	1.2 (0.8–1.5)
10	15	55	ASF T6-T12	4	-	-	5	109	1.2	79 (70–101)	2.6 (1.6–3.5)
11	2	9	ASF T12-L2	2	Conradi-Hünermann-Happle syndrome	-	1	34	0.3	58 (51–70)	1.4 (1.1–1.6)
12	10	22	ASF+PSF T2-S1	7	cerebral palsy, spastic tertraparesis	-	9	203	1.3	50 (39–56)	1.3 (1.1–1.5)
13	15	82	ASF+PSF T3-L3	7.5	-	fever, wound infection	30	103	2.5	64 (53–88)	1.1 (0.9–1.2)
14	16	54	ASF T7-T12	4	-	-	4	56	0.8	71 (63–82)	0.8 (0.6–1.3)
15	13	20	PSF T2-S1	8	spina bifida; Arnold Chiari type 2 malformation	peroperative anaphylactic shock to Venofundin	75	180	1.0	53 (33–78)	0.9 (0.8–1.1)
16	13	14	PSF T2-L5	4	Ullrich disease	-	41	129	0.3	59 (41–72)	0.7 (0.5–0.8)
17	9	16	PSF T3-L4	5	Pierre Robin Sequence; acampolic campomelic dysplasia	-	6	94	1.1	67 (57–93)	1.1 (0.9–1.4)
18	12	52	PSF T6-L1	5	-	UTI	6	58	2.8	68 (60–81)	0.9 (0.7–1.1)
19	16	55	PSF T5-L4	6.5	-	-	40	123	1.3	70 (58–87)	0.7 (0.6–1.0)
20	12	34	PSF L4-S1	6	spondylolisthesis	-	18	103	0.9	56 (49–73)	0.8 (0.7–0.9)

0 No: patient number in sequence of entrance to the study.

1 ASF: anterior spinal fusion; PSF: posterior spinal fusion.

2 IVH: intraventricular haemorrhage.

3 UTI: urinary tract infection.

4 these parameters are measured intra-operatively.

y: years, kg: kilograms, hr: hours, MAP: mean arterial pressure.

### Plasma I-FABP, I-BABP

**Figure 1:**
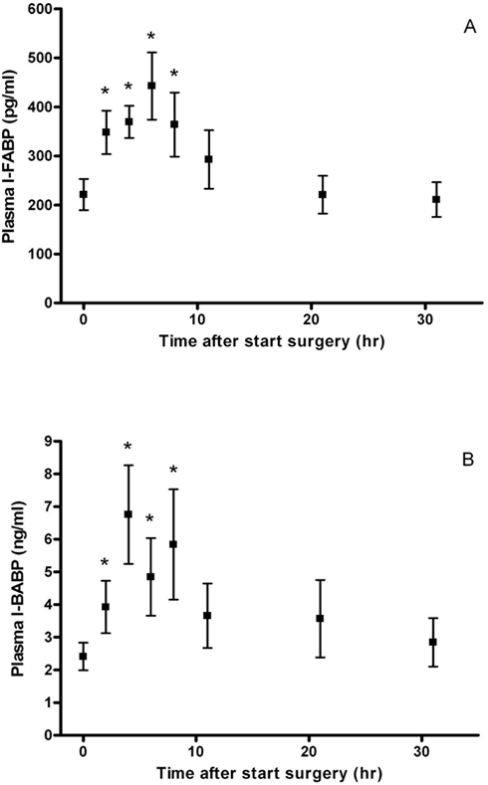
Time course of mean (SEM) plasma I-FABP (a) and I-BABP (b) levels in children undergoing spinal fusion surgery (n = 20). * p<0.05 vs. baseline values.

The plasma concentration of I-FABP increased rapidly after the initiation of surgery from a mean (SEM) baseline value of 221 (32) pg/ml shortly before start of surgery, under anaesthesia *(in-house mean normal value: 106 pg/ml, range: 41-336 pg/ml)* to 348 (44) pg/ml at 2 hours after the onset of surgery (p = 0.006) (Figure 1a). Thereafter, the mean plasma levels increased further to 369 (33) pg/ml (p<0.001) at 4 hours after initiation of surgery. The peak value of 443 (69) pg/ml (p<0.001) was reached at 6 hours after the start of surgery, which often represented the end of surgery. Thirteen patients showed an increase in plasma I-FABP levels of at least twofold during surgery; while 7 patients had relatively unchanged circulating I-FABP values. Plasma concentrations of I-FABP decreased towards baseline values from 2 hours after the end of surgery onwards.

Similar to the I-FABP levels, mean I-BABP plasma concentrations also increased significantly between 2 and 8 hours after start of surgery compared to baseline values in most of the patients (Figure 1b).

Since FABP are excreted by the kidneys, we evaluated whether high plasma values of FABP could be caused by impaired renal function. Diuresis during and after surgery was adequate (Table 1) and plasma creatinine values were not elevated, which indicates that elevation of plasma FABP was caused by enterocyte cell death.

### Urinary claudin-3

**Figure 2:**
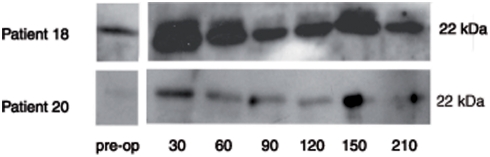
Time course of urinary claudin-3:creatinine ratio of two representative of the last five children undergoing spinal fusion surgery.

The urinary claudin-3:creatinine ratio immediately increased during the first 20 minutes of surgery. In the next 2 hours, the claudin-3:creatinine ratio remained high and thereafter a decrease towards preoperative values was detected (Figure 2).

### Gastric tonometry and mean arterial pressure in relation to intestinal damage

**Figure 3:**
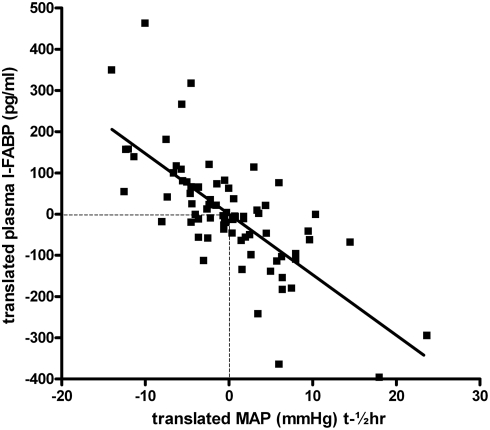
Translated values of plasma levels of circulating I-FABP and translated values of preceding systemic hypotension (MAP t-½hr) in children undergoing spinal fusion surgery were plotted. Circulating I-FABP correlated significantly negatively with MAP at ½ hour before blood sampling (n = 89, correlation: −0.726 (p<0.001). Translations of both variables are specific for an individual in such a way that all within-person means correspond to the zeros in the plot. In this way the variation of individual levels are cancelled and the pure association of both variables remains.

The very short circulating half-life of FABP (approximately 11 minutes) [27] allows to relate the presence of enterocyte cell damage with preceding systemic hypotension and gastric mucosal hypoperfusion. To this end within-person correlations were studied between circulating levels of I-FABP, I-BABP and intraoperative MAP at ½ hour before the blood sample was collected in which FABP concentration was measured, and P_r_CO_2_, P_r-a_CO_2_-gap at the same moment of blood sampling. Interestingly, plasma levels of I-FABP, I-BABP were significantly negatively correlated with MAP at ½ hour before blood sampling (correlation: −0.726 (p<0.001); −0.483 (P<0.001), respectively), indicating a relationship between enterocyte cell damage and preceding systemic hypotension (Figure 3, Table 2). Furthermore, circulating values of I-FABP correlated with gastric mucosal P_r_CO_2_ and P_r-a_CO_2_-gap measured at the same time points (correlation: 0.553 (p = 0.040) and 0.585 (p = 0.028), respectively), whereas no correlation was observed between plasma levels of I-BABP and P_r_CO_2_ or P_r-a_CO_2_-gap. These data show a clear association between the most prominent plasma marker for enterocyte cell death (I-FABP), hypotension and splanchnic hypoperfusion, assessed by gastric mucosal P_r_CO_2_ and P_r-a_CO_2_-gap.

**Table 2:** Within-person correlations between enterocyte cell damage and preceding systemic hypotension and gastric mucosal hypoperfusion.

*N*	MAP t-½hr (mmHg)	P_r_CO_2_ (kPa)	P_r-a_CO_2_-gap (kPa)
	*89*	*32*	*32*
**I-FABP (pg/ml)**	−0.726 (P<0.001)	0.553 (P = 0.040)	0.585 (P = 0.028)
**I-BABP (ng/ml)**	−0.483 (P<0.001)	−0.051 (P = 0.862)	−0.079 (P = 0.787)

Within-person correlations between enterocyte cell damage (plasma I-FABP and I-BABP) and preceding systemic hypotension (mean arterial pressure (MAP) at ½ hour before collection of the blood sample for FABP assessment) and gastric mucosal hypoperfusion (P_r_CO_2_, P_r-a_CO_2_-gap at the same moment of blood sampling). *N* = number of measurements.

### Complications

Six patients had nine early complications, including postoperative fever (n = 3), urinary tract infection (n = 2), pneumonia (n = 1), wound infection (n = 1), peroperative anaphylactic reaction to poly(O-2-hydroxyethyl)starch (Venofundin) (n = 1) and melaena of unknown origin (n = 1).

**Figure 4:**
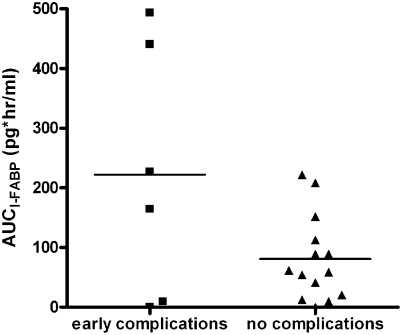
Area under the curve of plasma I-FABP values (AUC_I-FABP_) during surgery for six patients with early complications and 14 patients without complications. Mean AUC_I-FABP_ was significantly higher in patients with complications than in patients without complications (p = 0.032). The horizontal lines indicate the mean AUC_I-FABP_.

The mean AUC_I-FABP_ for the six patients with complications was 222 pg*hr/ml (range: 0–493 pg*hr/ml), while for patients without complications the mean AUC_I-FABP_ was 81 pg*hr/ml (range: 0–222 pg*hr/ml) (p = 0.032) (Figure 4). No significant changes were found in mean AUC_I-BABP_ during surgery between patients with and without complications (3.1 vs. 2.0 ng*hr/ml, p = 0.341).

## Discussion

The data showing an early increase of circulating FABP and urinary claudin-3, followed by rapid return towards baseline values, indicate that the patients suffered transient injury to the mature enterocytes and their tight junctions. Interestingly, similar kinetics of circulating FABP and urinary claudin-3 were found in all patients, regardless of extension of surgery or amount of blood loss. It remains to be established whether this insult is sufficient for a breakdown of the intestinal mucosal barrier, eliciting an inflammatory response and postoperative complications, as has been shown in animal studies [5], [8], [28]. Although this study was only set up to explore the development of intestinal mucosal cell damage during major non-abdominal surgery, a limited analysis of the postoperative course of patients with intestinal mucosal cell damage revealed that higher plasma levels of I-FABP were associated with a higher rate of postoperative complications. All possible complications are described as end point, because this type of surgery in relatively healthy children rarely results in important complications, including sepsis, MOF and death. Nevertheless, the described complications were associated with prolonged hospitalization. Taken together, this study did not prove causality that the observed gut barrier loss and inflammation are the inducing factors for SIRS and MOF. Additional work, enrolling more patients, including those likely to experience serious complications, is needed in order to fully unravel the sequelae of the observed gut barrier loss.

Our work is supported by three previous studies showing the temporary presence of intestinal villous cell damage, measured by increased urinary levels of I-FABP, in patients undergoing cardiac surgery with cardiopulmonary bypass (CPB) [29]–[31]. In line, the patients with high urinary I-FABP levels developed postoperative gastro-intestinal complications [29]. The use of CPB was shown to be responsible for alterations in blood flow with consequently intestinal mucosal hypoxia and villous tip ischemia [30], [31]. In our patients a similar influence of variation in blood flow on the provocation of intestinal villous cell injury was found, without the use of extracorporeal circulation.

Hypotension is often accepted to diminish blood loss and thereby facilitating surgical exposure and reducing the need for blood transfusions during e.g. oromaxillofacial, neurosurgery and major orthopaedic surgery [32], [33]. Peroperative hypotension is considered to be caused by hypovolaemia and/or anaesthetics. It is unlikely that the children undergoing spinal fusion repair were hypovolaemic, because fluids were administered adequately, which is reflected by positive fluid balance, low plasma lactate levels and sufficient diuresis. Therefore, anaesthetics are the major cause of low MAP. Propofol and sevoflurane, which were used in almost all children as anaesthetic agents have only minimal effects on cardiac output, but they decrease the systemic vascular resistance significantly, resulting in hypotension [34]. However, the fall in blood pressure together with prolonged surgery and anaemia potentially results in tissue hypoxia, represented by transient splanchnic hypoperfusion, impairment of hepatocellular integrity, renal dysfunction and visual loss because of optic nerve ischemia [32], [33], [35]. Our study shows for the first time the relation between accepted hypotension and the development of intestinal mucosal cellular damage in patients undergoing major non-abdominal surgery.

The clinical consequences of our findings are challenging. It is clear that major (non-abdominal) surgery, accompanied by accepted systemic hypotension aimed at minimizing intraoperative blood loss, can induce splanchnic mucosal hypoperfusion and gut barrier loss. While organ blood flow regulation is preserved over a wide range of MAP, organ perfusion becomes pressure dependent when the MAP decreases below a certain critical level (autoregulatory threshold). The autoregulatory threshold varies between different organs, the presence of diseases and age; little data are available on the autoregulatory threshold in children, and no studies report on the child intestinal autoregulation. We are currently performing a study in which haemodynamic optimization, aimed at normotension and flow-directed parameters, is intended during major surgery in order to prevent the development of intestinal damage.

The present study concerns relatively healthy children and young adolescents, who have an insignificant risk for important complications after major surgery. In line, studies with older patients undergoing surgery or trauma show that increasing age is one of the most crucial risk factors influencing adverse outcome [36], [37]. Therefore, we speculate that the loss of the intestinal mucosal barrier as observed in our study in relatively healthy children undergoing major non-abdominal surgery would have a larger effect on the development of postoperative complications in older patients. Collectively, these findings shed a new light on the potential role of intestinal barrier compromise during major surgery, which was adapted from numerous animal studies, but now reported in relatively healthy children and adolescents undergoing major non-abdominal surgery. Furthermore, we consider that these results indicate a need to re-examine currently accepted criteria of haemodynamic parameters in patients undergoing major surgery.
